# Anesthetic Considerations for Patients on Psychotropic Drug Therapies

**DOI:** 10.3390/neurolint13040062

**Published:** 2021-11-29

**Authors:** Monica W. Harbell, Catalina Dumitrascu, Layne Bettini, Soojie Yu, Cameron M. Thiele, Veerandra Koyyalamudi

**Affiliations:** 1 Department of Anesthesiology and Perioperative Medicine, Mayo Clinic, 5777 East Mayo Boulevard, Phoenix, AZ 85054, USA; Dumitrascu.Catalina@mayo.edu (C.D.); Bettini.Layne@mayo.edu (L.B.); Yu.Soojie@mayo.edu (S.Y.); Koyyalamudi.Veerandra@mayo.edu (V.K.); 2 Mayo Clinic Alix School of Medicine, Scottsdale, AZ 85259, USA; Thiele.Cameron@mayo.edu

**Keywords:** antipsychotic agents, psychotropic drugs, antidepressive agents, anesthesia, anesthetics, perioperative period, drug interactions

## Abstract

Psychotropic drugs are used in the treatment of psychiatric and non-psychiatric conditions. Many patients who are on psychotropic medications may present for procedures requiring anesthesia. Psychotropic medications can have dangerous interactions with drugs commonly used in anesthesia, some of which can be life-threatening. In this review, we describe the current anesthetic considerations for patients on psychotropic drug therapies, including antidepressants, antipsychotics, mood stabilizers, anxiolytics, and stimulants. The pharmacology, side effects, and potential drug interactions of the commonly prescribed psychotropic drug therapies with anesthetic agents are described. Further, we highlight the current recommendations regarding the cessation and continuation of these medications during the perioperative period.

## 1. Introduction

More than one billion people, or one in seven people, experience mental health or substance use disorders globally [1]. Psychotropic drugs are commonly prescribed in the treatment of psychiatric conditions and are increasingly being used off-label for treatment of Tourette’s syndrome [2], eating disorders, insomnia [3], and chronic pain [4]. It is estimated that one in six adults in the United States use psychotropic medications, with an even higher incidence in the elderly [5]. The most commonly used psychotropic medications are antidepressants, which are used by 12% of all US adults, followed by anxiolytics, sedatives, and hypnotics at 8.3% and antipsychotics at 1.6% [5]. Typically, patients take these medications on a long-term basis, with 84.3% [5] reporting that they have taken psychotropic medications for longer than three years.

Given the increase in prescription of psychotropic medications, many patients taking psychotropic medications may present for procedures requiring anesthesia. Psychotropic medications can have potentially dangerous interactions with drugs commonly used in anesthesia, some of which can be life-threatening. It is important for anesthesia professionals to have an up-to-date knowledge of psychotropic medications, their side effects, their interactions with commonly used anesthetic agents, and the management of these medications perioperatively. Further, the withdrawal of these psychotropic medications in the perioperative setting can have important anesthetic implications and should be considered when caring for patients on psychotropic medications.

In this review of the current literature, we describe the pharmacology, side effects, and potential drug interactions of the commonly prescribed psychotropic drug therapies with anesthetic agents. The following classes of psychotropic medications will be discussed, including antidepressants, antipsychotics, mood stabilizers, anxiolytics, and stimulants. We will also discuss the current recommendations regarding the cessation and continuation of these medications during perioperative period.

## 2. Methods

A review of the current literature from years 2000 to 2021 on PubMed and Google Scholar was conducted in April–June 2021, with an emphasis on literature published in the past 5 years. The following search terms were used: “Psychotropic drugs”, “Psychotropic medications”, “Psychoactive”, “Antidepressants”, “Antipsychotics”, “Mood stabilizers”, “Anxiolytics”, “Stimulants”, “Benzodiazepines”, “Barbiturates” AND “anesthetic implications” or “anesthetic considerations”, “anesthetic interactions”, or “Perioperative considerations” (in all combinations). For each selected article, the reference list was reviewed for additional sources.

## 3. Antidepressants

A significant number of patients undergoing surgery and receiving anesthesia are treated with antidepressant medications. These drugs include tricyclic antidepressants (TCAs), selective serotonin reuptake inhibitors (SSRIs), and serotonin–norepinephrine reuptake inhibitors (SNRIs), monoamine oxidase inhibitors (MAOIs), atypical antidepressants, and herbal medications. Possible side effects and interactions of these medications with anesthetics should be considered in the perioperative period (Table 1). Antidepressant medications are generally continued in the perioperative period to prevent relapse of symptoms and withdrawal [6] (Table 2).

### 3.1. Tricyclic Antidepressants 

TCAs were the primary treatment for depression for numerous years. They also have been shown to improve a variety of disorders including anxiety, pain syndromes, obsessive compulsive disorder, enuresis, insomnia, and cocaine withdrawal [7]. TCAs contain an iminodibenzyl (tricyclic) core, and the primary mechanism of action is through serotonin and norepinephrine reuptake inhibition [8]. Subtle differences in their structure result in significant variability in their physiological effect. For example, some TCAs have an effect primarily on serotonin reuptake, while others act primarily via norepinephrine [7]. Similarly, certain TCAs are highly anticholinergic (e.g., imipramine), while others are less so (desipramine) [8]. TCA metabolism occurs through the P450 system with variability in CYP enzyme inhibition [9]. 

Side effects of TCAs can be significant and vary depending on the specific drug. Anticholinergic effects are highest with amitriptyline and include sedation, urinary retention, constipation, prolonged gastric emptying, dry mouth, blurry vision, confusion, and delirium [6,7] (Table 1). As a result of antimuscarinic and quinidine-like Class I antiarrhythmic effects, TCAs can also have cardiac complications, such as direct myocardial depression, tachycardia, significant arrhythmias, prolongations of electrocardiogram (ECG) intervals, and alterations in contractility [6,7]. Due to the anticholinergic and cardiac depressant properties, drug overdose with TCAs is among the most dangerous [7]. TCAs can also cause orthostatic hypotension, lower the seizure threshold, and can lead to loss of libido [7]. Abrupt cessation of TCAs in the perioperative period can lead to cholinergic rebound withdrawal symptoms including malaise, rhinorrhea, and abdominal pain [7,10]. Given the significant side effect profile and lower tolerability, TCAs are no longer first-line antidepressants [8].

Perioperatively, special attention should be given to patients with pre-existing cardiac comorbidities (arrhythmias, conduction abnormalities, and ischemic heart disease), and a preoperative electrocardiogram should be completed given the risk for QT prolongation [6,11]. In patients with a history of seizures or renal failure, caution should be taken with the combination of TCAs with other seizure-threshold-reducing drugs, such as meperidine [11]. Similarly, serotonin syndrome may be precipitated when TCAs are combined with other serotonin-increasing medications such as phenylpiperidine opioids (meperidine, methadone, and fentanyl), tramadol, ondansetron, metoclopramide, metronidazole, second generation antipsychotics, and St. John’s wort [9]. The triad of serotonin syndrome consists of mental status changes such as agitation and confusion, autonomic instability including hyperthermia and hemodynamic variability, and neuromuscular abnormalities such as hyperreflexia and rigidity [9]. Due to the inhibition of norepinephrine reuptake, there is also a risk of an exaggerated response to indirect-acting vasopressors and sympathetic stimulation [6,11]. Given the sympathomimetic properties, ketamine, pancuronium, meperidine, and local anesthetics containing epinephrine should therefore be avoided in patients taking TCAs [6,12]. Hypotension is also possible through chronic catecholamine depletion and resultant unopposed anesthetic agent-induced cardiac depression [6]. A small dose of a direct-acting vasopressor, such as phenylephrine, is the primary treatment rather than indirect-acting vasopressors, such as ephedrine [6]. Lastly, patients taking TCAs may have increased minimal alveolar concentration (MAC) or total anesthetic requirements due to enhanced brain catecholamines. Conversely, when used in conjunction with centrally acting anticholinergic agents such as atropine and scopolamine, these patients are at increased risk of postoperative sedation and delirium [6]. 

### 3.2. Selective Serotonin Reuptake Inhibitors and Serotonin–Norepinephrine Reuptake Inhibitors 

SSRIs and SNRIs are often well-tolerated and frequently considered first-line treatment for depression [6]. They have also been used for anxiety and panic disorders, bulimia, post-traumatic stress disorder, and obsessive–compulsive disorder [6]. SSRIs include fluoxetine, sertraline, paroxetine, fluvoxamine, citalopram, and escitalopram. SSRIs inhibit the CYP-450 enzymes (least with citalopram and escitalopram), with minimal anticholinergic properties (the highest with paroxetine), sedative effects, and orthostatic hypotension. SNRIs include desvenlafaxine, duloxetine, milnacipran, and levomilnacipran. Venlafaxine inhibits CYP-450, while the active metabolite, desvenlafaxine, does not [10]. Due to both serotonergic and noradrenergic properties, SNRIs have also been used to treat pain and stress incontinence [7].

This group of antidepressants have a milder side effect profile compared to TCAs and MAOIs due to little or no anticholinergic or cardiac activity and have a decreased risk of fatal overdose. Primary side effects of SSRIs include headache, nausea, tinnitus, agitation, insomnia, and sexual side effects including erectile dysfunction and dysorgasmia [6,7] (Table 1). Side effects of SNRIs consist of hypertension secondary to norepinephrine reuptake inhibition, tachycardia, sexual dysfunction, mydriasis, urinary constriction, dry mouth, dizziness, and sedation [7,10]. SSRIs and SNRIs are typically continued perioperatively, as rapid discontinuation may result in withdrawal symptoms of dizziness, anxiety, agitation, dizziness, malaise, dysphoria, and rarely extrapyramidal symptoms [7].

Though the clinical significance is unclear, it is important to note that SSRIs and SNRIs can result in abnormal bleeding secondary to alterations of platelet serotonin levels [13]. The metaanalysis by Laporte et al. found a 36% increased bleeding risk in these patients [13]. Several mechanisms appear to contribute to this risk including blockage of platelet calcium, nitric oxide synthase inhibition, decreased platelet factors, and reduced platelet activation secondary to decreased expression of membrane receptors [13]. Sertraline and citalopram have the lowest risk of surgical bleeding when used with warfarin [7]. As noted above, serotonin syndrome can result with these medications when they are used in excess or when they are combined with other serotonergic drugs. Special consideration should also be given to blood pressure control given the possible hypertensive effects of SNRIs. Citalopram may increase the QT interval at high doses, and venlafaxine may have a greater risk of arrhythmia in overdose [7]. Lastly, levels of various drugs including antiarrhythmic drugs, benzodiazepines, and neuromuscular blocking medications may be increased due to inhibition of cytochrome-P450 enzymes [12].

### 3.3. Monoamine Oxidase Inhibitors

Although once considered a first-line treatment for depression, MAOIs are now reserved for resistant depression due to their toxicity profile and dietary restrictions [6,7,10]. MAOIs act by inhibiting oxidative deamination of amines, thereby increasing concentrations of MAO substrates including norepinephrine, serotonin, and dopamine [6,10]. Isoenzyme Type A (MAO-A) is selective for serotonin, dopamine, and norepinephrine, while isoenzyme Type B (MAO-B) is selective for dopamine and phenylethylamine [6]. Among the nonselective irreversible MAOIs are phenelzine, isocarboxazid, and tranylcypromine, which are indicated in refractory depression [6,10].

Side effects of MAOIs are a result of changes in neurotransmitter concentrations, as well as anticholinergic effects [10]. These include agitation, orthostatic hypotension, muscle spasms, seizures, paresthesia, elevated liver enzymes, urinary retention, jaundice, nausea, insomnia, sexual dysfunction, and sympathomimetic effects such as tachycardia and tremor [6,7,10,11] (Table 1). One of the most concerning complications of MAOI use is hypertensive crisis. MAOIs reduce the monoamine degradation of tyramine, resulting in excess tyramine that displaces stored monoamines (dopamine, norepinephrine, and epinephrine) from the presynaptic vesicles and leads to hypertensive crisis. This can occur after the ingestion of foods containing tyramine, and thus, patients must avoid tyramine-containing foods, such as cheese, wine, fava beans, and avocado [10]. Furthermore, medications containing phenylpropanolamine, phenylephrine, dextromethorphan, pseudoephedrine, as well as drugs that have the potential to increase serotonin leading to serotonin syndrome should be avoided [6,7]. MAOIs are often continued perioperatively due to a long tapering period, possible increased suicidal ideation, and withdrawal symptoms of agitation, cognitive dysfunction, and headache [8,12].

Intraoperatively, blood pressure should be monitored carefully given the risk of orthostatic hypotension and hypertensive crisis with an exaggerated response to sympathomimetics. The placement of an arterial line can be considered for patients with other comorbidities (e.g., cardiac) at the discretion of the anesthesiologist. Indirect-acting sympathomimetic drugs and ketamine should be avoided [9,12]. Small doses of direct-acting vasopressors such as phenylephrine should be used if hypotension occurs [6,12]. Meperidine is contraindicated in patients taking MAOIs due to the risk of serotonin syndrome [6,9,12]. Caution should also be used with the administration of opioids, particularly the phenylpiperidine opioids discussed previously [6]. Multimodal analgesia should be utilized, with a regional block (local anesthetic without epinephrine) when appropriate to avoid opioid-induced hyperpyrexia in patients on MAOIs [11,12]. Phenelzine has been shown to decrease plasma cholinesterase and can therefore prolong neuromuscular blockade with succinylcholine and mivacurium [6]. Lastly, MAC requirement is increased secondary to increased circulating norepinephrine, and it is recommended that greater depth of anesthesia is maintained given the risk of sympathetic stimulation [12].

### 3.4. Other Antidepressants

Numerous other atypical antidepressants may be used by patients presenting to the operating room. Atypical antidepressants include second generation SNRIs, norepinephrine and dopamine reuptake inhibitors (NDRIs), serotonin antagonist and reuptake inhibitors (SARIs), or combined reuptake inhibitors and receptor blockers (CRIBs), such as bupropion, trazodone, nefazodone, venlafaxine, vilazodone, and vortioxetine [12]. Nefazodone inhibits cytochrome P450 3A4 isoenzymes and therefore can lead to QT prolongation and tachyarrhythmias and has been associated with liver failure [6]. The noradrenergic and specific serotonergic antidepressant (NaSSAs) mirtazapine has less sexual dysfunction compared to TCAs, SSRI/SNRIs, and MAOIs. Mirtazapine is advantageous as an antiemetic given its 5HT_3_ receptor antagonism and is useful for insomnia given its antihistamine effects [8]. Due to a high affinity for histamine 1 receptors at low doses, mirtazapine has a paradoxical effect of increased in sedation with lower compared to higher doses [14]. Side effects of mirtazapine include increased appetite and weight gain, lipid disturbances, and dizziness [8]. Several drugs have similar interactions and precautions as MAOIs listed above, with the most common side effects being hypertension and hyperpyrexia [12]. Similarly, combined use with MAOIs is contraindicated with these medications.

### 3.5. Herbal Supplements

Various herbal supplements are increasingly being used for the treatment of depression, and 32% of ambulatory surgery patients report using herbal supplements [15]. A list of the more commonly used herbal supplements for psychiatric conditions are included in Table 3 [16,17,18,19,20,21]. These medications are often not included on the patient’s medication list but may interact with anesthetics, affect the efficacy of prescription medications, and have other consequences perioperatively [6,11,15]. In general, these supplements should be discontinued at least 2 weeks before surgery, though more specific discontinuation timelines have been suggested for individual supplements [11]. While a variety of herbal supplements are available to patients for depression and anxiety, perioperative implications of St. John’s wort, Kava, and Valerian are discussed here. St. John’s wort functions primarily through the inhibition of serotonin, norepinephrine, and dopamine reuptake and weakly as a MAO inhibitor [11,16,17]. It is an inducer of the hepatic CYP 3A4 enzyme system and may therefore decrease the concentrations of other drugs, such as midazolam, and can lead to delayed emergence [9,11]. Due to the inhibition of neurotransmitter reuptake and slight MAOI, patients taking St. John’s wort are at risk of serotonin syndrome and thus other drugs that increase serotonin, MAOIs, and meperidine should be avoided in these patients [6,11]. It is recommended that St. John’s wort is discontinued five days prior to surgery. Kava is an herbal supplement that has been used for a variety of reasons including relaxation and insomnia via potentiation of γ-aminobutyric acid (GABA) [11,15]. Through inhibition of sodium and calcium channels, kava has been shown to decrease systemic vascular resistance and can lead to intraoperative hypotension. It also inhibits cyclooxygenase, leading to a possible decrease in renal blood flow and platelet dysfunction [11,15]. Kava may increase the effects of benzodiazepines and lead to increased sedation postoperatively and also has the potential to cause hepatic injury [15]. It is recommended that kava be discontinued 23 h before surgery and earlier when the surgery performed may result in compromise of blood flow to the liver [11]. Valerian is used in assisting with sedation and in the treatment of insomnia, likely through GABA mediation [11]. It and can lead to prolonged sedation when in conjunction with anesthetics. Importantly, abrupt discontinuation of valerian in patients who are dependent in the setting of long-term use may lead to acute withdrawal symptoms similar to benzodiazepine withdrawal [11]. It is unclear when valerian should be discontinued prior to surgery in non-dependent patients; however, a gradual decrease in dose is recommended for patients that have physical dependence [11]. In the setting of valerian withdrawal, benzodiazepines can be utilized to mitigate symptoms [11]. 

## 4. Antipsychotics

Antipsychotic drugs (APDs) are a group of drugs primarily used for the treatment of schizophrenia and bipolar disease. APDs are classified primarily into two groups, typical and atypical antipsychotic drugs. This distinction depends on their ability to cause extrapyramidal side effects and tardive dyskinesia, which is less likely in the atypical group [22].

The predominant mechanism of action of typical APDs is by the blockade of postsynaptic brain dopamine D_2_ receptors. These drugs were effective in treating positive symptoms associated with schizophrenia such as hallucinations and delusions, however, were relatively ineffective in treating the negative symptoms, which include blunted affect, decreased motivation, asociality, reduced experience of pleasure, and reduced quantity of words spoken [23]. Up to 60% of patients with schizophrenia may have prominent clinically relevant negative symptoms that require treatment [23].

Second generation or atypical APDs have a lower incidence of extrapyramidal side effects. Most atypical APDs have a higher affinity for serotonin 5HT_2_ receptor binding than the dopamine D_2_ receptors, which is in contrast with typical APDs that preferentially bind to the dopamine D_2_ receptors [22]. This might account for the reduced incidence of extrapyramidal side effects seen with the use of atypical APDs.

Absorption after oral intake varies considerably among the various APDs. Most of these drugs undergo extensive first-pass metabolism by the liver, resulting in variable bioavailability. There is marked variation in protein binding and volume of distribution among the patient population, leading to difficulty in predicting serum drug levels. APDs are primarily metabolized by the cytochrome P450 system and therefore are subject to significant drug interactions in the presence of diminished liver function and with medications that may induce or inhibit enzymes of the cytochrome P450 system [24]. One notable exception is the atypical APD, paliperidone, which is primarily excreted unchanged via the kidneys, and only a small fraction of the drug is inactivated by hepatic enzymes. Cytochrome P450 CYP3A4 and CYP2D6 enzymes have been implicated in the metabolism of paliperidone [25]. Clearance of the various APDs ranges from 6 h to up to 4 days. Genetic pleomorphisms in CYP2D6 has been found to be associated with a significant variation in the rates of clearance of some APDs [26].

APDs generally have additional affinities for other neurotransmitter receptor subtypes [27]. These include other serotonin (5-HT_1A_, 5-HT_2C_, 5-HT_6_, and 5-HT_7_), dopamine (D_1_, D_3_, and D_4_), histamine (H_1_), muscarinic (M_1_, M_2_, M_3_, M_4_, and M_5_), and adrenergic receptors (*α*_1_ and *α*_2_) [27] with resultant anticholinergic, hypotensive, sedative, and metabolic side effects. The incidence of the side effects varies considerably across the large number of typical and atypical APDs. For example, atypical antipsychotics have metabolic side effects with the most common being Type 2 Diabetes Mellitus, particularly with clozapine and olanzapine [28] (Table 1).

Both typical and atypical APDs have been found to be associated with an increased incidence of sudden cardiac death. These drugs block repolarizing potassium currents and prolong the QT interval potentially leading to ventricular arrythmias and cardiac arrest [29]. Torsade de pointes has been reported with the use of both typical and atypical APDs. The typical APDs associated with the greatest risk of QT interval prolongation are thioridazine, haloperidol, chlorpromazine, haloperidol, and pimozide [30]. Among atypical APDs, iloperidone, quetiapine, and ziprasidone have been shown to cause clinically significant increases in QTc [30]. Caution should be advised with the concomitant use of other drugs that have the propensity to prolong the QT interval. Preoperative evaluation should include an electrocardiogram to determine the presence of any QT prolongation.

APDs by virtue of alpha-adrenergic blockade effects can result in orthostatic hypotension [31]. This may be more prevalent in those patients with autonomic dysfunction, hypovolemia, and concomitant vasodilatory antihypertensive medication use. Among typical antipsychotic drugs, orthostatic hypotension is most common with thioridazine and chlorpromazine. Atypical APDs commonly associated with orthostatic hypotension include clozapine, iloperidone, quetiapine, and paliperidone.

Chlorpromazine and thioridazone have high levels of histamine H_1_ receptor antagonism and can be highly sedating [32]. Atypical APDs (except pimavanserin) cause sedation to a varying extent due to histamine H_1_ receptor-blocking effects [33].

Both typical and atypical APDs (highest risk with clozapine) have been found to reduce the seizure threshold but have not been associated with new onset seizures [34,35]. Caution should be used with the concomitant administration of other drugs that may reduce the threshold for seizures.

Neuroleptic malignant syndrome (NMS) is a life-threatening emergency associated with the use of antipsychotic agents. Both typical and atypical APDs have been implicated, though it is more commonly seen with formerly called neuroleptic agents such as haloperidol and fluphenazine. The most important risk factor is a prior history of NMS. NMS is characterized by the presence of mental status changes, rigidity, fever, and dysautonomia. NMS can occur after the initial single dose of the drug or may manifest many years after treatment with the same drug at the same dose. The pathogenesis of NMS is unknown and diagnoses is made by the presence of clinical signs while on APDs and exclusion of other conditions. Differential diagnoses include serotonin syndrome and malignant hyperthermia (Table 4). Treatment involves cessation of the APD, supportive therapy, and, in more severe cases, dantrolene, bromocriptine, amantadine, and benzodiazepines may be considered [36]. Electroconvulsant therapy has also been shown to be effective in cases refractory to medical treatment [36].

Anticholinergic side effects are seen with both typical and atypical APDs to a varying degree. Among the atypical APDs, these side effects are more common with chlorpromazine but very infrequently seen with risperidone. Symptoms include dry mouth, decreased salivation, decreased bronchial secretions (risk for bronchial mucus plugging), pupillary dilation (risk of angle closure glaucoma), tachycardia (which could lead to angina or myocardial infarction), constipation, inability to empty the bladder, and central effects such as memory loss and confusion [37].

Significant increase in the incidence of confusion and agitation was reported when APDs were discontinued for 72 h versus those patients who continued APDs until the day of surgery [38]. Therefore, routine cessation of APDs prior to surgery is not recommended despite the propensity for side effects as described above.

### 4.1. Mood Stabilizers

Mood stabilizers are prescribed for patients diagnosed with bipolar disorder, which affects about 2% of the world’s population [39]. Bipolar disorder is a group of severe and chronic conditions that include Bipolar I, characterized by a syndromal, manic episode and bipolar II, characterized by a hypomanic episode and a major depressive episode [40]. To stabilize these mood swings, patients can be on monotherapy or prescribed a combination of medications. As mood stabilizers can have a significant impact on anesthesia, the anesthetic considerations for the most commonly prescribed mood stabilizers will be described, including lithium, valproic acid, carbamazepine, and lamotrigine.

### 4.2. Lithium

Lithium is the gold standard treatment due to its antimanic, antidepressant, and antisuicide effects [40]. The mechanism of action for lithium is poorly understood. One proposed mechanism is that as an alkali metal and a monovalent cation, lithium mimics sodium and enters cells during depolarization [41]. This results in reduction in release of neurotransmitters both in the central and in the peripheral nervous system [41]. More recent work suggests that lithium has a neuroprotective factor by inhibiting NMDA receptor [41]. Another hypothesis is that lithium causes GSK3 inhibition and inositol depletion by inhibiting IMPase [39].

Although lithium is the treatment of choice, it has a narrow therapeutic window with optimal serum levels around 0.6–1.2 mmol/L [42]. The dose usually ranges from 600–1200 mg daily, but variation can occur depending on weight or symptoms [42]. When serum levels exceed 1.5 mmol/L, lithium toxicity occurs, resulting in confusion, sedation, muscle weakness, tremors, and slurred speech [43]. As lithium is renally excreted, dehydration, diuretics, and renal impairment can exacerbate the risk of toxicity. Given that perioperative hemodynamic instability can adversely affect renal function, it is recommended to discontinue lithium prior to surgery. The half-life of lithium is 24–37 h, and, therefore, lithium should be discontinued 72 h before surgery [43]. Lithium does not have any withdrawal effects once stopped [43]. If lithium is continued, medications that alter renal clearance should be avoided. Non-steroidal anti-inflammatory drugs can increase lithium levels on average by 10–25% [44]. Further, stimulation of urine production with thiazide diuretics should be avoided as it can reduce renal clearance of lithium [44].

Perioperatively, an electrocardiogram should be closely monitored as sinus node dysfunction, atrioventricular block, T-wave changes, hypotension, and ventricular irritability can occur with toxicity. In addition, train-of-four neuromonitoring is important, as lithium can prolong neuromuscular blockade [43]. Due to lithium-blocking brainstem release of norepinephrine, epinephrine, and dopamine, there is a reduction of MAC requirements [43].

### 4.3. Other Mood Stabilizers

To treat acute manic episodes, valproic acid (VPA) is often prescribed [45]. VPA is both an anticonvulsant and mood stabilizer. It enhances the inhibitory effect of gamma-aminobutyric acid (GABA), reduces repetitive neuronal firing, and decreases the inhibition and excitation within neuronal networks [45]. VPA can be continued perioperatively, but if it is discontinued, it does not have any withdrawal effects [41]. As VPA is highly plasma-protein-bound, it can result in an increase in the free concentration of other highly plasma-protein-bound drugs. Past studies have shown the propofol dose might be decreased in patients taking VPA [46,47]. Another perioperative concern in patients taking VPA is coagulopathy. VPA decreases platelet count and lowers Factor VII, Factor VIII, fibrinogen, and protein C levels [48].

Carbamazepine is also effective in treating acute manic episodes [40]. It acts by preventing repetitive firing by blocking inactivated sodium channels [41]. Carbamazepine can be continued perioperatively; however, carbamazepine is an inducer of the cytochrome p450 (Cyp450) system. Therefore, the effects of medications metabolized by the Cyp450 system can be reduced, including nondepolarizing amino-steroid neuromuscular blocking agents [41]. Patients taking carbamazepine will have significantly shorter duration of neuromuscular blockade, necessitating more frequent or higher doses of neuromuscular blocking agents. Coagulopathy is also a concern with carbamazepine as it can cause thrombocytopenia, leukopenia, and aplastic anemia [49]. Oxcarbazepine is a second-generation anti-epileptic medication that can be used to treat bipolar disorder. It is a weaker inducer of hepatic enzymes compared to carbamazepine but can still be clinically significant [50].

Few medications have been shown to be effective in preventing both mania and depression. Lamotrigine, technically an anticonvulsant, is prescribed for maintenance treatment of bipolar, but it is better at treating and preventing bipolar depression than mania. The mechanism of action may be due to inhibiting sodium and calcium channels in the presynaptic neurons, thereby stabilizing the neuronal membrane [51]. It decreases glutamate release and enhances GABA release. As it reduces glutamate release, ketamine’s dissociative anesthetic effect could be reduced [52].

Although bipolar disorder patients can be prescribed various antidepressant, antipsychotic, and antiepileptic medications to control their symptoms, the most common prescribed medications are lithium, valproic acid, carbamazepine, and lamotrigine. VPA, carbamazepine, oxcarbazepine, and lamotrigine can be continued in the perioperative period [53]. Lithium, however, should be discontinued due to increased risk of toxicity when renal clearance is compromised.

## 5. Anxiolytics

After antidepressants, anxiolytics are the second most-prescribed psychotropic medications. Although patients were once commonly prescribed barbiturates for anxiety, this class of medications has largely been replaced by benzodiazepines. Both classes of psychotropic medications can have important anesthetic considerations.

### 5.1. Benzodiazepines

Benzodiazepines act in the central nervous system (CNS) by binding to the GABA-A receptor and subsequently increasing the frequency of opening of the associated chloride channel. The potentiation of the chloride current in the CNS results in the hyperpolarization of neurons and reduced excitability. Benzodiazepines are highly lipid-soluble and rapidly enter the CNS, resulting in their rapid onset of action [54,55]. The main effects of these medications include sedation, anterograde amnesia, anxiolysis, and anti-convulsant activity [56]. Benzodiazepines may produce myocardial depression and hypotension when co-administered with opioids. Benzodiazepines decrease the ventilatory response to carbon dioxide; however, this is typically clinically insignificant unless combined with other respiratory depressants. In the brain, benzodiazepines reduce cerebral blood flow and intracranial pressure. The metabolism of benzodiazepines occurs in the liver via microsomal oxidation and glucuronide conjugation, and the metabolites are chiefly excreted in the urine [54,55].

Clinically, benzodiazepines have a variety of uses including anxiety, panic disorders, insomnia, muscle spasms, alcohol withdrawal, and as abortive therapy for seizures. However, these medications are typically reserved for short term or adjuvant use due to concerns for drug dependence, tolerance, withdrawal, and potential for abuse [6,24,57,58]. In the perioperative setting, benzodiazepines are used for preoperative anxiolysis, sedation, induction of anesthesia, and suppression of seizure activity [55,59]. Common side effects include sedation, cognitive impairment, psychomotor impairment, and, in severe cases, respiratory depression [12,24]. Given the high risk of cognitive impairment with benzodiazepine use in the elderly, its use should be avoided in those individuals older than 65 years of age [60]. Post-operative delirium is a serious complication following anesthesia, with the highest incidence being in elderly patients and patients with cognitive impairment. Premedication with benzodiazepines preoperatively is associated with a higher incidence of post-operative delirium (odds ratio of 3.0) with a dose-dependent relationship (each mg of midazolam administered is associated with an increased risk of postoperative delirium of up to 8%) [61,62]. Therefore, these medications should be avoided in patients who are known to be at an increased risk of post-operative delirium [62].

Perioperatively, benzodiazepines have the potential to interact with other commonly used medications for the induction and maintenance of anesthesia. Propofol, a frequently used medication for the induction and maintenance of anesthesia, reduces clearance of IV midazolam by 37% via inhibition of CYP3A4. Similarly, fentanyl, frequently used in the perioperative period, decreases midazolam clearance by 30% [56]. Furthermore, benzodiazepines decrease the MAC requirement of volatile anesthetics by as much as 30% [54]. In addition to pharmacokinetic interactions, there are important pharmacodynamic drug interactions to be considered perioperatively. For instance, propofol and midazolam may act synergistically for hypnosis and eliminating movement in response to noxious stimulation. This likely is due in part to the similar mechanism of action of these two medications via the GABA-A receptor complex. Additionally, the interaction between benzodiazepines and opioids is considered synergistic [56,63].

Overall, acute versus chronic use of benzodiazepines has different implications for anesthetic requirements. Acute use decreases anesthetic requirements, while chronic use, such as in patients with benzodiazepine substance abuse, increases anesthetic requirements [6]. Despite these various drug interactions, it is recommended that patients who are preoperatively on benzodiazepine treatment continue their medications perioperatively [64]. As benzodiazepines are one of the most frequently prescribed drugs worldwide and benzodiazepine misuse is a growing global public health problem with approximately 2% of the population misusing benzodiazepines, patients should carefully be asked about their prescribed and unprescribed benzodiazepine use history [65,66,67]. Further, anesthesia providers should pay close attention to the patient’s preoperative medication list, including herbal supplements. Supplements such as kava, St. John’s wort, and grapefruit juice may interact with benzodiazepines, as well as medications that inhibit cytochrome P450 enzymes, as many benzodiazepines are substrates of CYP3A4 or CYP219 [12,24,56].

It is also important to recognize that benzodiazepine withdrawal can occur after a single dose or with chronic use [12]. Benzodiazepine withdrawal is clinically manifested by anxiety, insomnia, and tremors [64]. The abrupt cessation of benzodiazepines in patients who have been taking them for more than a month can lead to life-threatening seizures, delirium, and even death [66]. Therefore, benzodiazepines should not be abruptly stopped preoperatively [66]. Treatment of benzodiazepine withdrawal consists of symptomatic care and tapered administration of benzodiazepines [12,24].

### 5.2. Barbiturates

Barbiturates act in a similar manner to benzodiazepines in that they act in the CNS by binding to a different site on the GABA-A receptor, potentiating the chloride current through the associated channel. However, in contrast to benzodiazepines, these medications increase the duration of channel opening. Clinical uses for barbiturates include reducing intracranial pressure (ICP), neuroprotection from focal ischemia, induction of anesthesia, and rapid sequence intubation. However, propofol has largely replaced barbiturates as the more frequent choice for anesthesia induction [54]. Barbiturates are also used in the critical care setting to treat refractory status epilepticus if benzodiazepines and propofol have failed [55]. The chemical structure of the medication has implications for drug activity and clinical uses. For example, the phenyl group at the C_5_ carbon of phenobarbital confers anticonvulsant activity. In contrast, the methyl group in methohexital is not anticonvulsive, making this medication useful for anesthesia in electroconvulsive therapy (ECT) [54]. Benzodiazepines have largely replaced barbiturates in their use for anxiolysis and sedation perioperatively [59]. Most barbiturates undergo hepatic metabolism, except for thiopental, which is eliminated by renal excretion.

As with benzodiazepines, acute barbiturate use will decrease anesthetic requirements, while chronic use increases anesthetic requirements [6]. Compared to benzodiazepines, barbiturates result in a greater degree of respiratory depression and hypotension. Hypotension principally occurs due to peripheral vasodilation. However, despite this decrease in blood pressure following bolus administration, cardiac output is typically maintained by a compensatory tachycardia and increase in myocardial contractility from baroreceptor compensatory mechanisms. Caution should be taken when administering bolus doses of barbiturates in situations where the baroreceptor response may be blunted or absent, such as in hypovolemia, cardiac tamponade, beta-adrenergic blockade, congestive heart failure, and cervical radiation in patients with history of head and neck cancer [68]. In these instances, cardiac output and blood pressure may fall dramatically due to a decreased ability to compensate for the effects of peripheral vasodilation [54,55]. Other clinic effects include cerebral vasoconstriction resulting in a reduction in ICP, reduction of cerebral oxygen demand, and reduction in hepatic and renal blood flow [54]. Barbiturates increase the production of porphyrins though stimulation of aminolevulinic synthetase and therefore should not be administered in patients with acute intermittent porphyria [55]. Another contraindication is in patients with status asthmaticus, because barbiturates incompletely depress airway responses to laryngoscopy and intubation and may lead to bronchospasm [54,69].

Similar to benzodiazepines, barbiturate withdrawal can result in life-threatening seizures. Other withdrawal symptoms include nervousness, tremor, agitation, and hypotension, which may develop 2–8 days following discontinuation of the medication [69]. Barbiturate overdose manifests with sedation, coma, respiratory depression, and hypotension. In contrast to benzodiazepine overdose, which can be reversed with flumazenil [12,54,56], there is no specific antidote for barbiturates, and overdose is managed with supportive care [70].

## 6. Stimulants

Central nervous system stimulants comprise an increasingly abundant drug class with both medicinal and recreational uses. Prescription stimulants are generally used to treat narcolepsy, attention deficit and hyperactivity disorders, and depression. Stimulants are used off-label or recreationally for their effects of euphoria, alertness, excitement, and appetite suppression. Recent data suggest that up to 6.6% of adults in the United States used prescription stimulants in 2016 [71], while the prevalence of recreational use of stimulants, such as cocaine, “ecstasy”, and amphetamines, in North America in 2017 ranged from 0.9–2.1% [72]. Whether prescribed or used recreationally, some patients using stimulants will inevitably require anesthesia, and it is therefore important to understand their anesthetic implications.

Commonly prescribed stimulants include mixed amphetamines, methylphenidate, and modafinil. Amphetamines (e.g., Adderall^®^) act as sympathomimetics by inhibiting reuptake of dopamine, norepinephrine, and serotonin in the cortex, motor nuclei, and reticular-activating system [73]. They are also thought to promote displacement of these neurotransmitters into the synaptic cleft [74]. Methylphenidate (Ritalin^®^) is a milder central nervous system stimulant, which acts similarly by inhibiting reuptake transporters for norepinephrine and dopamine [73]. Modafinil acts via a mechanism that continues to evade explanation but likely exerts its predominant wakefulness-promoting effects by inhibiting norepinephrine and dopamine transporters, or possibly by increasing the release of histamine, glutamate, and serotonin [74]. Stimulants historically have been prescribed for weight loss (e.g., “aminorex” and “fen-phen”) but have since been largely removed from the market for unfavorable cardiovascular risk–benefit profiles, including the development of pulmonary arterial hypertension and valvular heart disease [75]. 

The variety of recreational stimulants is vast, and ranges from plants (e.g., Khat) to lab-designed drugs (e.g., “ecstasy”). Some of the more commonly encountered recreational stimulants include cocaine, methamphetamine, and MDMA (3-methoxy-4,5-methylenedioxyamphetamine or ecstasy). Each has their own idiosyncrasies; however, to varying extents, all stimulants act via the modulation of norepinephrine, serotonin, and dopamine neurotransmitters. Acutely, stimulants cause feelings of profound euphoria and increased energy. Repeat or excessive dosing can result in psychosis, anxiety, and central nervous system irritability, occasionally resulting in seizures. Cardiovascular effects can include tachyarrhythmia, peripheral blood vessel constriction, hypertension, angina, myocardial infarction, and cerebral vascular accident [76]. Withdrawal symptoms include fatigue, irritability, insomnia, and paranoia, among others. There is a low likelihood of severe morbidity or mortality from withdrawal as can be seen with other substances such as alcohol.

There is growing evidence to support the uninterrupted use of prescribed stimulant medication throughout the perioperative period, when used as prescribed [77]. Even patients who use cocaine, if there is no acute intoxication, can likely undergo a safe general anesthetic [78]. Patients should be encouraged to discontinue recreational stimulant use prior to elective surgery.

It is worth noting that patients on long-term stimulants may have depleted endogenous catecholamine stores and may be resistant to sympathomimetic medication, thereby requiring direct-acting vasoconstrictors if indicated [76]. In the setting of acute stimulant intoxication, volatile anesthetic requirements will be increased. Invasive blood pressure monitoring should be considered with the potential for dysrhythmia and hemodynamic lability. Cocaine intoxication carries unique potential complications, including platelet dysfunction and coronary artery vasospasm [76]. Hypertension and tachycardia are common and can be particularly amplified during laryngoscopy and intubation. There is traditional teaching that suggests beta blocker use in cocaine toxicity can exacerbate coronary vasospasm and lead to myocardial injury. This “unopposed alpha stimulation” theory of myocardial injury appears to be a rare event and the complications attributed to it are likely just a result of the deleterious effects of cocaine [79]. All things considered, it may be best to rely on nitric oxide-mediated vasodilators, calcium channel blockers, and nonselective beta blockers (e.g., labetalol), for managing hemodynamic lability in cocaine intoxication. Chronic stimulant abuse can result in left ventricular hypertrophy and dilated cardiomyopathy [80].

Acute MDMA toxicity, in addition to the usual signs and symptoms described above, may present with severe hyponatremia from large-volume free water intake and increased antidiuretic hormone levels in the setting of serotonergic activity. This can lead to cerebral edema and seizures [81]. Hyperthermia and rhabdomyolysis may also be present; it is unclear if dantrolene or clozapine are helpful beyond supportive measures.

## 7. Discussion

Given the widespread use of psychotropic medications worldwide [5], it is imperative for anesthesia providers to maintain up-to-date knowledge of the pharmacology, side effects, and potential anesthetic implications of psychotropic medications. In the preoperative evaluation, anesthesia providers should ask patients about any history of psychiatric illness and conduct a thorough psychotropic medications use history. Anesthesia providers should keep in mind that patients may be taking psychotropic medications for off-label uses, such as the treatment of chronic pain [12]. This thorough medication history must include herbal supplements with psychotropic effects and those used recreationally. As patients on psychotropic medications may present with cognitive impairment [12], it is important to involve family members or caretakers for collateral history and even potentially to provide consent in severe cases. The cognitive impairment due to psychotropic drugs can mask serious medical conditions such as stroke, drug overdose, and diabetic ketoacidosis [12]. Family members and caretakers can be crucial in identifying these mental status changes from baseline.

The most common side effects, drug interactions, and anesthetic considerations for each class of psychotropic medications are summarized in Table 1. Anesthesia providers must consider the potential drug interactions that can occur when certain psychotropic medications are combined with commonly used medications in anesthesia. Each anesthetic should be tailored to the individual patient, keeping in mind the potential for drug interactions. For example, in patients receiving TCAs and MAOIs, a regional anesthetic may be used; however, local anesthetics with epinephrine should be avoided as it may precipitate a hypertensive crisis.

Other serious complications can arise with the combination of psychotropic drugs and anesthetic agents, such as seizures with the combination of tricyclic antidepressants and meperidine or hypertensive crisis when indirect-acting vasopressors are used with MAOI. There is even the potential for life-threatening serotonin syndrome with the interaction of antidepressants with phenylpiperidine opioids and commonly used antiemetics such as ondansetron and metoclopramide. It can be challenging to distinguish serotonin syndrome from other life-threatening conditions that occur perioperatively, such as neuroleptic malignant syndrome, malignant hyperthermia, and cholinergic crisis. Symptoms and distinguishing characteristics of these conditions are highlighted in Table 4.

There can be significant consequences to withdrawal from psychotropic medications and thus, most psychotropic medications can and should be continued perioperatively (Table 2). A notable exception is lithium, which ideally should be discontinued 72 h prior to surgery. However, the decision to discontinue a psychotropic medication should be carefully considered weighing the risk of drug interaction with anesthesia, risk of withdrawal symptoms, and risk of relapse or worsening mental health. This decision should be made preoperatively in conjunction with the patient’s psychiatrist and mental health support team. If a psychotropic medication is discontinued preoperatively, a plan for exactly when this can be restarted postoperatively should be decided, with special consideration if oral medications are not an option postoperatively due to the surgery.

As altered mental status is not an uncommon occurrence after receiving anesthesia, it is critical for anesthesia providers to have evaluated the patient’s baseline mental status prior to anesthesia. Anesthesia providers should involve family members and caretakers in helping to identify these changes in mental status. Further, anesthesia providers should consider if the psychotropic medication itself, withdrawal from the medication, or drug interaction of the psychotropic medication with anesthetic agents are contributing to the altered mental status.

In conclusion, the management of patients on psychotropic medications in the perioperative period involves a multidisciplinary approach and should include the patient, their family, and their mental health support team. Most psychotropic medications should be continued perioperatively and only discontinued after careful discussion with patient and mental health providers and with a definitive plan of when to resume the medication. Anesthesia providers must obtain a thorough history of any psychotropic drug use and tailor the anesthetic to minimize any potential drug interactions.

## Figures and Tables

**Table 1:** Side effects, drug interactions, and anesthetic considerations for psychotropic drugs.

Drug Class	Side Effects	Drug Interactions/Precautions with Anesthesia
Antidepressants		
Tricyclic antidepressants	Anticholinergic (sedation, urinary retention, constipation, prolonged gastric emptying, dry mouth, blurry vision, confusion, delirium), direct myocardial depression, tachycardia, arrhythmias, ECG interval prolongation, alterations in contractility, orthostatic hypotension, lower seizure threshold, sexual dysfunction	Caution in patients with preexisting arrhythmias and conduction abnormalities, preoperative EKG Seizure: avoid meperidine Serotonin syndrome *: avoid phenylpiperidine opioids (meperidine, methadone, and fentanyl), tramadol, ondansetron, metoclopramide, metronidazole, second generation antipsychotics, and St. John’s wort Hypertension: avoid sympathetic stimulation and ketamine Sedation: avoid additional anticholinergics (atropine and scopolamine) Increased MAC requirements Multimodal analgesia and regional anesthesia without epinephrine when possible
SSRI/SNRI	Abnormal bleeding, headache, nausea, tinnitus, agitation, insomnia, sexual dysfunction, hypertension, tachycardia, mydriasis, urinary constriction, dry mouth, dizziness, sedation	Abnormal bleeding Serotonin syndrome Hypertension QT prolongation Increased duration of action of benzodiazepine and neuromuscular blocking medications
Monoamine oxide inhibitors	Agitation, orthostatic hypotension, muscle spasms, seizures, paresthesia, urinary retention, dry mouth, jaundice, nausea, diarrhea, constipation, headache, dizziness, drowsiness, insomnia, sexual dysfunction, tachycardia, tremor, hypertension	Hypertensive crisis: avoid indirect-acting vasopressors (ephedrine), ketamine, local anesthetics containing epinephrine Serotonin syndrome: meperidine contraindicated Phenelzine: prolonged neuromuscular blockade with succinylcholine Multimodal analgesia and regional anesthesia when possible
Second generation antidepressants	Hypertension, hyperpyrexia	Serotonin syndrome
**Antipsychotics**	Anticholinergic, orthostatic hypotension, QT prolongation, sudden cardiac death, sedation, lowers seizure threshold, Neuroleptic Malignant Syndrome	Preoperative EKG to evaluate QT, caution with other seizure-threshold-lowering drugs
**Mood stabilizers**		
Lithium	Toxicity with levels > 1.5 mmol/L; confusion, sedation, muscle weakness, tremors, and slurred speech; EKG changes of sinus node dysfunction, AV block, T wave changes	Prolongs neuromuscular blockade, possible decrease in anesthetic requirements
Valproic acid	Thrombocytopenia, decreases Factor VII, Factor VIII, fibrinogen, and protein C	Highly protein-bound, so free concentration of high plasma-protein-bound drugs can be increased such as propofol
Carbamazepine	Cytochrome P450 inducer	Cytochrome p450 inducer so medications metabolized by this system can be affected
Lamotrigine		Decrease glutamate release → reduced dissociative effect of ketamine
**Anxiolytics**	Sedation, cognitive impairment, psychomotor impairment, respiratory depression, anterograde amnesia.	Drug interactions: Kava, St. John’s Wart, and grapefruit juice, as well as medications that inhibit cytochrome P450 enzymes Anesthesia precautions: benzodiazepines decrease the MAC requirement of volatile anesthetics, synergistic effect with propofol and opioids.
**Stimulants**	Euphoria, anxiety, insomnia, psychosis, seizures, tachyarrhythmia, peripheral blood vessel constriction, hypertension, angina, myocardial infarction, and cerebral vascular accident.	Endogenous catecholamine depletion and resistance to sympathomimetic drugs; consider direct-acting vasoactive medications.

SSRI, Selective serotonin reuptake inhibitors; SNRI, serotonin–norepinephrine reuptake inhibitors; mmol/L, millimole/liter; EKG, electrocardiogram; AV block, atrioventricular block. * To prevent serotonin syndrome, avoid the following medications: phenylpiperidine opioids (meperidine, methadone, and fentanyl), tramadol, ondansetron, metoclopramide, metronidazole, second generation antipsychotics, and St. John’s wort.

**Table 2:** Summary of perioperative recommendations.

Drug Class	Continuation/Discontinuation Recommendation Perioperatively
**Antidepressants**	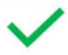
**Antipyschotics**	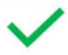
**Mood stabilizers**	
**Lithium**	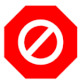 Discontinue 72 h before surgery
**Valproic acid**, **carbamazepine**, **lamotrigine**	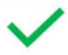
**Anxiolytics**	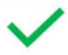
**Stimulants**	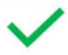

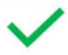
 = safe to continue perioperatively.

**Table 3:** Common herbal supplements used as psychotropic drugs and their side effects.

Herbal Supplement	Indications	Potential Side Effects
Ginkgo biloba	Cognitive disorders, dementia, erectile dysfunction	Inhibition of platelet-activating factor
Kava	Anxiety, sedation	Delayed emergence, hepatic injury, inhibits CYP3A4 and CYP2E1, hypotension, decreased renal blood flow, platelet dysfunction
Melatonin	Insomnia	Sedation, confusion, hypothermia, immunosuppression, delayed emergence
N-acetyl cysteine	Depression, obsessive compulsive disorder	None
Omega-3 fatty acids	Depression, bipolar disorder, psychotic disorders, borderline personality disorder, attention-deficit disorders	GI upset, mania in patients with bipolar disorder
S-adenosyl methionine (SAMe)	Depression	Mania in patients with bipolar, GI upset, insomnia, anorexia, dry mouth, sweating, dizziness, nervousness
St. John’s wort	Anxiety, insomnia, depression	Dry mouth, dizziness, serotonin syndrome when combined with SSRIs or meperidine, increases rate of absorption of methadone with potential for opioid withdrawal, mania in patients with bipolar, constipation, phototoxicity, prolong effects of anesthesia and delayed emergence
Valerian	Insomnia	Inhibits CYP3A4, blurry vision, dystonia, hepatotoxicity, dose-dependent sedative and anxiolytic effects, caution use with benzodiazepines and opiates, delayed emergence, benzodiazepine-like withdrawal
Zinc	Depression	Nausea

**Table 4:** Differential diagnosis of neuroleptic malignant syndrome and serotonin syndrome.

	Causes/Precipitating Factors	Symptoms/Signs	Treatment
**Neuroleptic Malignant Syndrome**	Anti-dopaminergic drugs (e.g., antipsychotics, metoclopramide) Abrupt cessation of dopaminergic drugs (Levodopa)	-Muscle rigidity, hyperthermia, ↑CK, myoglobinuria, ↑HR, diaphoresis, ↑secretions, tremor, AMS, urinary incontinence. -**chorea**, **akinesia**, **opisthotonos**, **trismus**, **blepharospasm**, **and oculogyric crisis**	-Discontinue offending agent (or restarting drug if dopaminergic drug withdrawal) -Aggressive hydration if ↑CK -If severe, bromocriptine or dantrolene. -Cool patient
**Malignant Hyperthermia**	Autosomal dominant inheritance. Triggered by succinylcholine and volatile anesthetics.	Hyperthermia, muscle rigidity, ↑CK, myoglobinuria, ↑HR, ↑**CO_2_** **production**, **tachypnea**, sympathetic nervous system overactivation (tachyarrhythmias and ↑BP).	-Discontinue offending agents. -Hyperventilation with 100% O_2_ -Immediate administration of dantrolene. -Cool patient if fever -Treat acidosis and electrolyte abnormalities.
**Serotonin syndrome**	Precipitated by serotonergic drugs	-Classic triad: AMS, autonomic hyperactivity, and neuromuscular abnormalities. -Mild: hypertension, ↑HR, diaphoresis, tremor, **myoclonus**, **mydriasis**, **hyperreflexia**. -Moderate: mild features plus hyperthermia, **hyperactive bowel sounds**, **agitation**, **and hypervigilance**. -Severe cases: above features plus worsening hyperthermia, muscle rigidity, and **delirium**	-Discontinuation of serotonergic drugs. -Mild: benzodiazepines, IV fluids -Moderate: telemetry, serotonin antagonist (cyproheptadine). -Severe cases: ICU admission with sedation, paralysis, and intubation.
**Cholinergic crisis**	Excessive use of AChEI (pyridostigmine, neostigmine), organophosphate poisoning	-Muscarinic receptor stimulation: salivation, ↓HR, lacrimation, urinary frequency/urgency, **diarrhea**, **GI cramping**, **emesis**, **and miosis**. -Cholinergic receptor stimulation: **muscle fasciculations**, **weakness**, **respiratory muscle weakness**, ↑HR, ↑BP. -CNS stimulation: seizures, coma, **ataxia**, **slurred speech**, agitation.	-Discontinue offending agent. -Secure airway if GCS < 8, excess secretions, respiratory muscle weakness -Atropine to reverse muscarinic effects. -Pralidoxime if organophosphate poisoning

CK, creatinine kinase; AMS, altered mental status; AChEI, anticholinesterase inhibitors; GCS, Glasgow Coma Scale. **Bolded symptoms are the distinguishing feature.**

## Data Availability

The data presented in this study are available in the article.
